# Sulfation of Arabinogalactan Proteins Confers Privileged Nutrient Status to Bacteroides plebeius

**DOI:** 10.1128/mBio.01368-21

**Published:** 2021-08-03

**Authors:** Jose Munoz-Munoz, Didier Ndeh, Pedro Fernandez-Julia, Gemma Walton, Bernard Henrissat, Harry J. Gilbert

**Affiliations:** a Biosciences Institute, The Medical School, Newcastle University, Newcastle upon Tyne, United Kingdom; b Microbial Enzymology Group, Department of Applied Sciences, Northumbria University, Newcastle upon Tyne, United Kingdom; c Department of Food and Nutritional Sciences, Whiteknights, University of Reading, Reading, United Kingdom; d DTU Bioengineering, Technical University of Denmark, Lyngby, Denmark; e King Abdulaziz University, Department of Biological Sciences, Jeddah, Saudi Arabia; Brigham and Women's Hospital/Harvard Medical School

**Keywords:** *Bacteroides*, human microbiota, arabinogalactan, glycan-degrading enzymes, microbial ecology, privileged nutrient, sulfatases

## Abstract

The human gut microbiota (HGM) contributes to the physiology and health of its host. The health benefits provided by dietary manipulation of the HGM require knowledge of how glycans, the major nutrients available to this ecosystem, are metabolized. Arabinogalactan proteins (AGPs) are a ubiquitous feature of plant polysaccharides available to the HGM. Although the galactan backbone and galactooligosaccharide side chains of AGPs are conserved, the decorations of these structures are highly variable. Here, we tested the hypothesis that these variations in arabinogalactan decoration provide a selection mechanism for specific *Bacteroides* species within the HGM. The data showed that only a single bacterium, B. plebeius, grew on red wine AGP (Wi-AGP) and seaweed AGP (SW-AGP) in mono- or mixed culture. Wi-AGP thus acts as a privileged nutrient for a *Bacteroides* species within the HGM that utilizes marine and terrestrial plant glycans. The B. plebeius polysaccharide utilization loci (PULs) upregulated by AGPs encoded a polysaccharide lyase, located in the enzyme family GH145, which hydrolyzed Rha-Glc linkages in Wi-AGP. Further analysis of GH145 identified an enzyme with two active sites that displayed glycoside hydrolase and lyase activities, respectively, which conferred substrate flexibility for different AGPs. The AGP-degrading apparatus of B. plebeius also contained a sulfatase, BpS1_8, active on SW-AGP and Wi-AGP, which played a pivotal role in the utilization of these glycans by the bacterium. BpS1_8 enabled other *Bacteroides* species to access the sulfated AGPs, providing a route to introducing privileged nutrient utilization into probiotic and commensal organisms that could improve human health.

## INTRODUCTION

The human large bowel is resident to trillions of bacteria that play a pivotal role in the health and nutrition of their host (1, 2). The major nutrients available to this microbial community, defined as the human gut microbiota (HGM), are dietary polysaccharides, which are not degraded by endogenous intestinal enzymes, and host glycans (3, 4). Reflecting their dependence on complex carbohydrates bacteria within the HGM, particularly those belonging to the *Bacteroidetes* phylum, express large numbers of carbohydrate-active enzymes, or CAZymes (5, 6). Manipulating the HGM through dietary or nutraceutical strategies offers opportunities for maximizing the health benefits of this microbial community. Given the importance of polysaccharides as a major nutrient for the HGM, understanding complex glycan utilization by this ecosystem is an essential prerequisite to successful dietary intervention. Consequently, there have been numerous studies exploring the mechanisms by which specific members of the HGM utilize selected glycans (7–12) (for reviews, see references 13 and 14). The *Bacteroides* spp., in general, produce surface endo-acting glycoside hydrolases or polysaccharide lyases that initiate the degradation of specific polysaccharides. The resultant oligosaccharides, imported into the periplasm, are degraded by an extended repertoire of CAZymes, leading to the generation of the monosaccharide components of these glycans. The genes encoding specific glycan-degrading systems are coregulated by the target polysaccharide and are organized into genomic regions termed polysaccharide utilization loci, or PULs (15). In addition to orchestrating the synthesis of the requisite CAZymes, PULs also encode the outer member transport protein, termed SusC, and surface binding proteins, such as SusD homologs (16) and the glycan sensor/regulator (17).

Arabinogalactan proteins (AGPs) are a ubiquitous feature of plant polysaccharides available to the HGM. In highly processed plant-derived components of the diet, such as red wine, the levels of complex plant-derived carbohydrates, such as AGPs and rhamnogalacturonan II (RGII), are particularly elevated (18). This is because during fermentation, yeast is able to utilize simple carbohydrates but not complex glycans such as AGPs and RGII. The glycan component of AGPs (comprising 90% of these glycoproteins) comprises a backbone of β-1,3-galactose units that are decorated with β-1,6-galactooligosacchrides and arabinofuranose units. The β-1,6-galactan side chains are often decorated with a range of sugars such as rhamnose, glucuronic acid, and additional arabinose units (19). Previous studies have shown that the utilization of arabinogalactans between organisms is dependent on the degree of polymerization (DP) of the glycan (20). Indeed, the capacity to grow on complex arabinogalactans with a high DP is restricted to a very small number of *Bacteroides* spp. These keystone organisms generated oligosaccharides that were utilized by bacteria capable of growing only on simple small arabinogalactans (20). Based on these observations, we hypothesize that the variable structures of arabinogalactans provide a selection mechanism for specific *Bacteroides* spp. within the HGM.

To test the hypothesis proposed above, we evaluated the capacity of our collection of *Bacteroides* spp. isolated from the HGM to grow on an AGP isolated from wine (Wi-AGP). The data showed that only a single bacterium, B. plebeius, was capable of growing on the glycan in mono- or mixed culture. Wi-AGP can thus act as a privileged nutrient for a *Bacteroides* spp. that has acquired the capacity to utilize marine and terrestrial plant glycans. Analysis of the enzymes required to depolymerize Wi-AGP demonstrated that a sulfatase was critical to the utilization of this glycan, consistent with the observed sulfation of this glycan. Furthermore, the substrate flexibility of an AGP-specific CAZyme was mediated through two distinct active sites, displaying glycoside hydrolase and polysaccharide lyase activities, respectively, which, uniquely, are located in a single catalytic module.

## RESULTS AND DISCUSSION

### Wi-AGP is utilized exclusively by B. plebeius.

**FIG 1:**
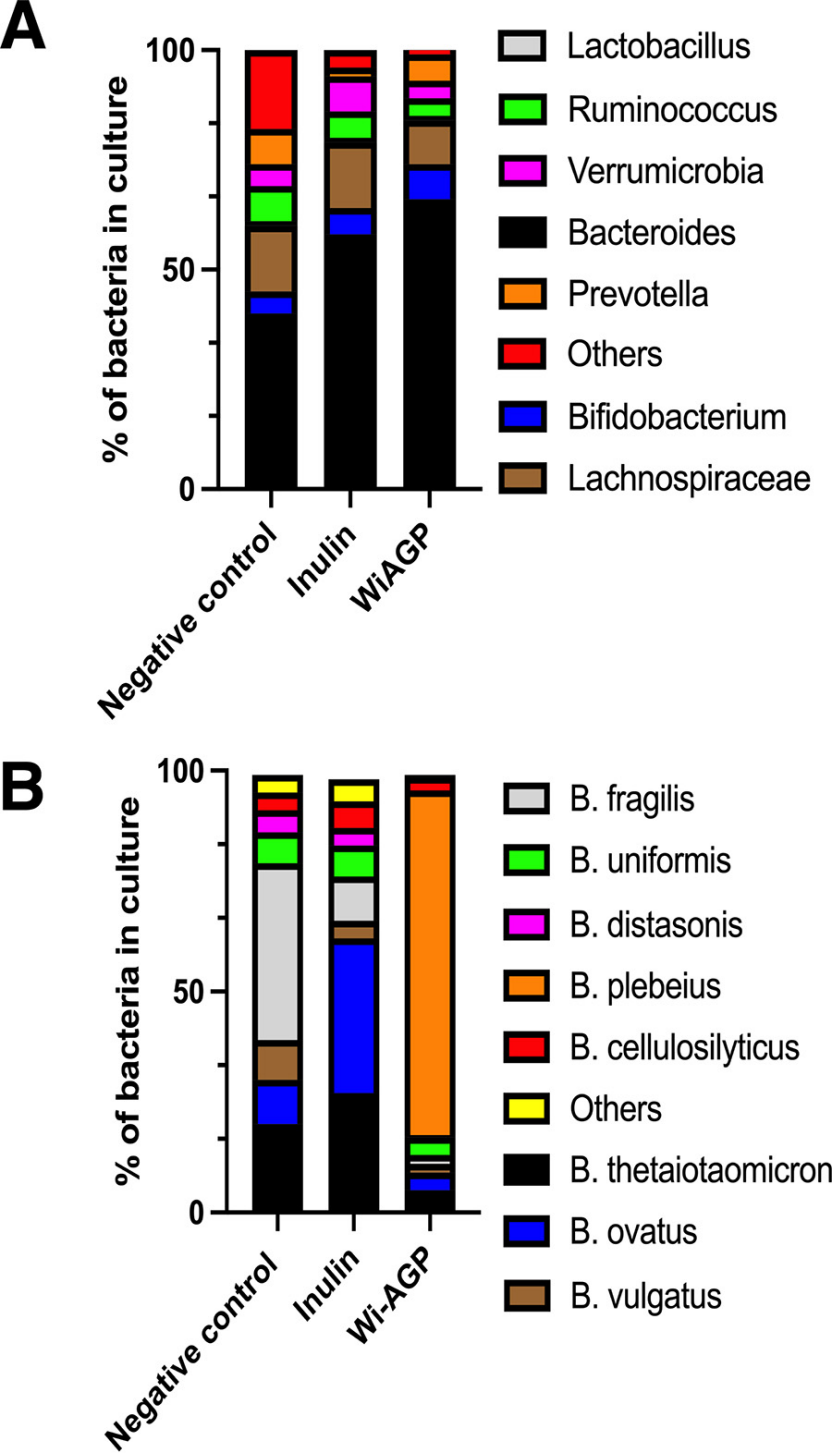
Proportion of bacterial genera and species in models of the HGM grown on different carbon sources. The HGM in a model colon was cultured in minimal media containing no carbon source (negative control), inulin, or red wine AGP (Wi-AGP). After 24 h, DNA was extracted from these cultures and subjected to metagenomics sequencing to determine the proportion of bacteria present. (A) Proportion of the major genera found in the HGM; (B) percentage of *Bacteroides* species from this microbial community. The numerical percentages of organisms in panels A and B are given in Table S1 in the supplemental material.

Wi-AGP was purified from red wine by gel filtration chromatography, and its capacity to act as a growth substrate for our collection of HGM *Bacteroides* spp. was evaluated. The data showed that only B. plebeius grew on Wi-AGP (Table 1). To further explore Wi-AGP bacterial utilization, the ability of the glycan to act as a growth substrate in batch cultures mimicking the distal colon inoculated with a fecal sample (comprises the HGM) was assessed (Fig. 1). The proportion of bacteria in the original HGM and the ecosystem cultured on inulin or Wi-AGP was evaluated by sequencing the V4 of 16S rRNA molecules. V4 is one of the highly variable regions of rRNA, and its sequence provides information on the genus and species of its bacterial origin (21). In the uncultured HGM B. plebeius, rRNA was not detected among the 162,000 V4 sequences obtained, in which 40% of the sequences were from the *Bacteroides* genus. This demonstrates that B. plebeius, if present, comprises an extremely small proportion of the HGM. When the HGM was cultured on inulin, there was an increase in the proportion of the *Bacteroides* and *Verrucomicrobia* genera; however, B. plebeius was again not detected among the 204,000 sequences analyzed. In the HGM grown on Wi-AGP, 64% of the 540,000 sequences obtained were from *Bacteroides* species. Significantly, ∼80% of the *Bacteroides* sequences encoded B. plebeius rRNA molecules, which equates to 50% of the total HGM. The data showed that Wi-AGP strongly selects for the growth of B. plebeius within the *Bacteroides* genus and in models of the HGM, and thus, this glycan is a privileged nutrient for the bacterium.

**TABLE 1:**
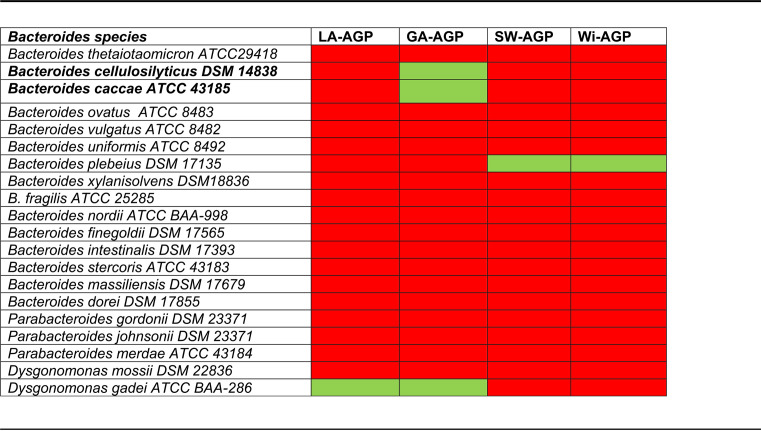
HGM-derived *Bacteroides* species cultured on LA-, GA-, SW- and Wi-AGP^*a*^

a Cells colored green and red indicate, respectively, growth or no growth of the bacteria. The data for larchwood (LA-AGP) and gum arabic AGP (GA-AGP) were published previously (20).

10.1128/mBio.01368-21.1 TABLE S1 Percentage of bacterial genera or species in cultured HGM. Download Table S1, DOCX file, 0.01 MB. Copyright © 2021 Munoz-Munoz et al. 2021 Munoz-Munoz et al. https://creativecommons.org/licenses/by/4.0/ This content is distributed under the terms of the Creative Commons Attribution 4.0 International license.

### B. plebeius utilizes a sulfated AGP.

**FIG 2:**
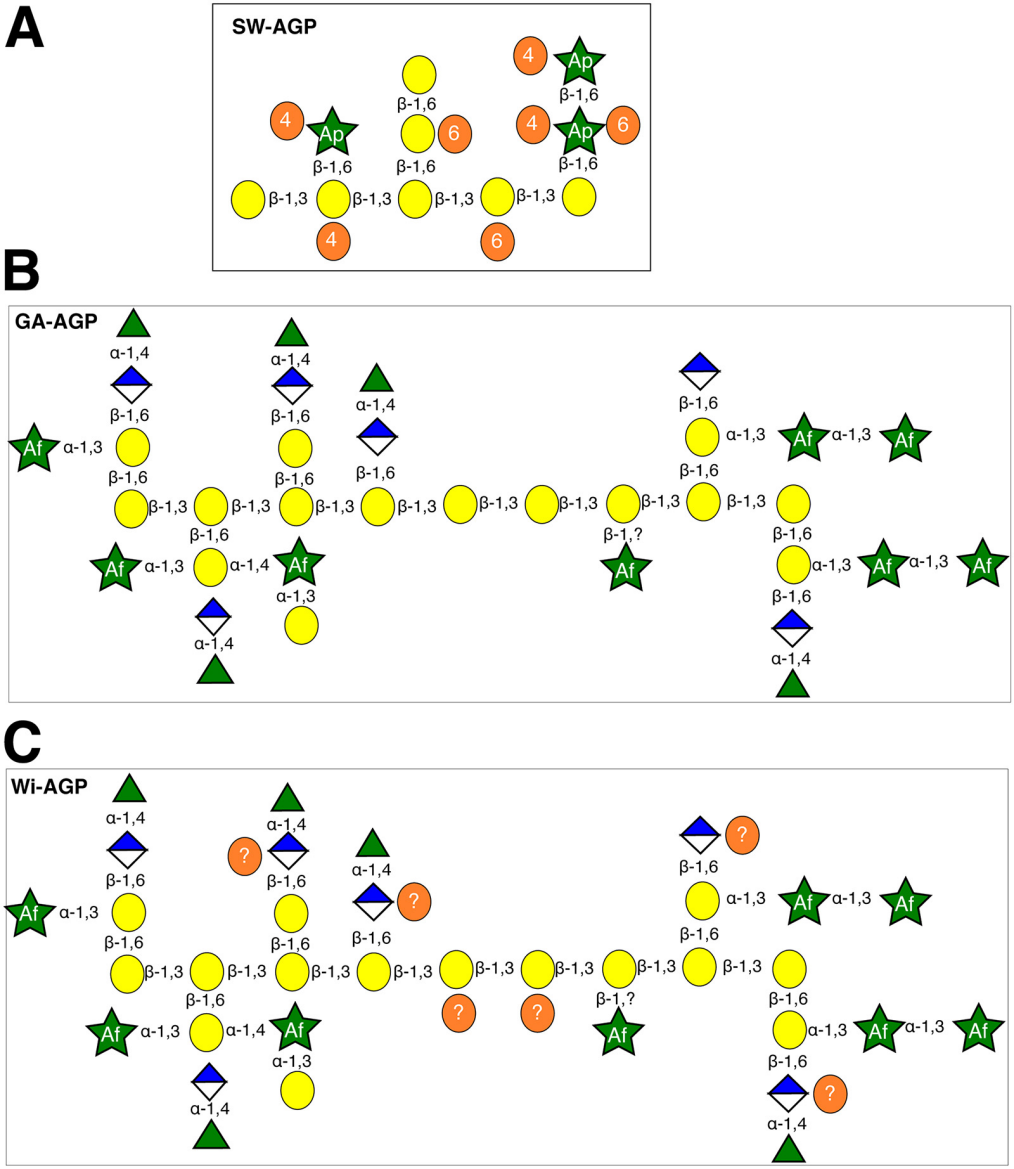
Structures of selected arabinogalactans. Structures of SW-AGP (A), GA-AGP (B), and Wi-AGP (C). The proposed structure of Wi-AGP is based on the observed activities of the GH145 polysaccharide lyase, the observation that BpS1_8 releases sulfate from Wi-AGP and that desulfation is required for cleavage of the β-1,3-galactan backbone by the GH43_24 exo-β-1,3-galactanase BpGH43_24. The precise position of the sulfates is unknown, hence the insertion of a question mark.

B. plebeius is an unusual member of the HGM in that it has acquired the capacity to utilize a marine polysaccharide, porphyran, which is a major carbohydrate in the seaweed (red algae) Porphyra yezoensis (22). Marine algal polysaccharides ae often highly sulfated, exemplified by porphyran and AGP from the green alga Codium fragile (SW-AGP) (23). To assess whether the capacity of B. plebeius to utilize Wi-AGP reflects its ability to desulfate glycans, growth of our collection of HGM-derived *Bacteroides* species on SW-AGP was assessed. The data (Table 1) showed that only B. plebeius was able to utilize SW-AGP. The structure of SW-AGP (Fig. 2) comprises a simple glycan in which the β-1,3-galactan backbone, the β-1,6-galactooligosacchrides, and the arabinose side chains are all sulfated (24). We propose that the unique capacity of B. plebeius to utilize SW-AGP and Wi-AGP reflects the ability of the bacterium to desulfate these polysaccharides.

**FIG 3:**
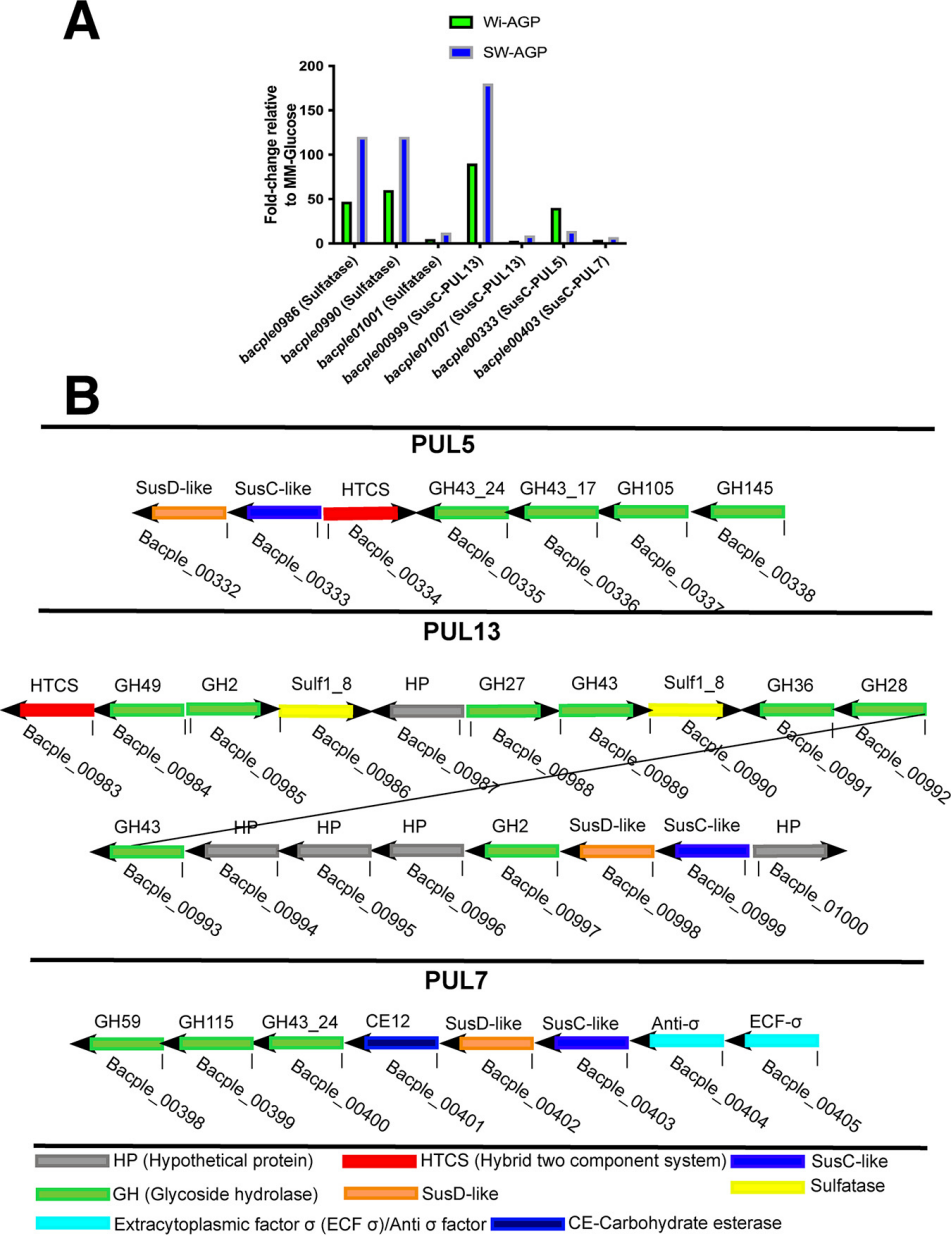
PULs upregulated by Wi-AGP and SW-AGP. (A) Cultures of B. plebeius were grown on SW-AGP (green bars) and Wi-AGP (blue bars) to mid-logarithmic phase, at which point RNA was extracted and subjected to quantitative reverse transcription-PCR (RT-PCR) using primers to amplify the genes shown. (B) Schematic of B. plebeius PULs upregulated by growth on the AGPs.

To test the sulfatase hypothesis, PULs in the B. plebeius genome that are likely to orchestrate AGP degradation were identified using the PUL database (www.cazy.org/PULDB_new/) (25) and knowledge of the predicted activities of the CAZymes encoded by these loci. Within the CAZy database, glycoside hydrolases and polysaccharide lyases are grouped into sequence-based families and subfamilies (5). In many instances, the substrate specificity of these enzymes can be predicted by their assignment to a CAZy family. Inspection of the 42 PULs within the genome of B. plebeius revealed three loci, PUL5, PUL7, and PUL13, that contain genes encoding enzymes likely to contribute to AGP degradation. Examples include enzymes in glycoside hydrolase family 2 (GH2), GH27, GH43, and GH145, which contain a range of arabinofuranosidases, arabinopyranosidases, galactosidases, glucuronidases, and rhamnosidases (5). To evaluate whether Wi-AGP and SW-AGP activate transcription of the three PULs, B. plebeius was cultured on the two glycans, and the mRNA levels of the cognate SusC genes, encoding outer membrane glycan transporters, were determined. These genes were selected, as they are expressed at high levels compared to other sequences within PULs. Furthermore, as they are always upregulated in an actively transcribed PUL, they are excellent indicators of loci that are switched on by a specific nutrient (26). The data (Fig. 3) showed that *bacple00403*, *the susC* gene of PUL7, was not activated by either AGP, indicating that this locus does not contribute to the observed growth on the two glycans. Significant transcription of *susC* from PUL5 and PUL13 was observed, demonstrating that both loci contribute to AGP degradation. The expression of *susC* from PUL13, however, was higher in response to SW-AGP than Wi-AGP. In contrast, transcripts of the equivalent gene in PUL5 were elevated in Wi-AGP cultures compared to when SW-AGP was the growth substrate, where upregulation of the locus was very modest. The observed differential transcription of the two AGP PULs by SW-AGP and Wi-AGP suggests that PUL5 is essential for the degradation of the wine glycan, while PUL13 contributes to the deconstruction of both polysaccharides. PUL5 encodes GH43_24 (Fig. 3), a subfamily that contains exclusively exo-β-1,3-galactanases (20). Of more significance, however, is the presence of the genes encoding GH145 and GH105 enzymes that are required to remove l-rhamnose-α-1,4-d-glucuronic acid (Rha-GlcA) disaccharides that cap some of the side chains in Wi-AG (see below); these structures are not present in SW-AGP. It is not clear why the expression of the locus is more pronounced in SW-AGP cultures but may reflect the concentration of the inducing ligands generated from the marine glycan.

**FIG 4:**
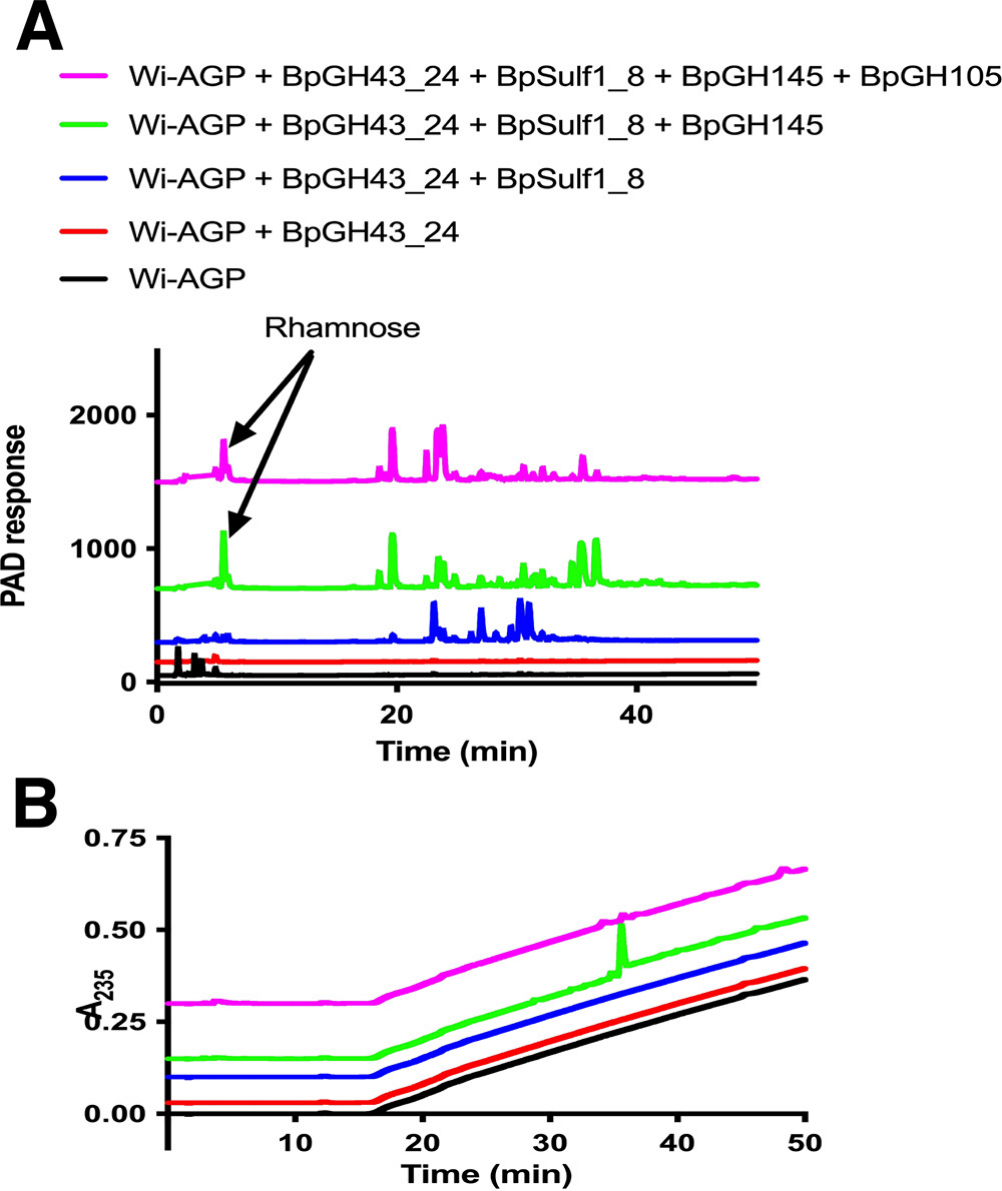
HPAEC analysis of the activity of selected enzymes encoded by PUL5 and PUL13. Wi-AGP was at 5 mg/ml for all reactions. Enzyme concentration was 1 μM. Reaction mixtures were incubated for 16 h in 20 mM sodium phosphate buffer, pH 7.0, containing 150 mM NaCl buffer. The data shown are representative of three independent replicates. Oligosaccharides generated were monitored by pulsed amperometric detection (PAD) (A) or UV absorbance at 235 nm (B).

**FIG 5:**
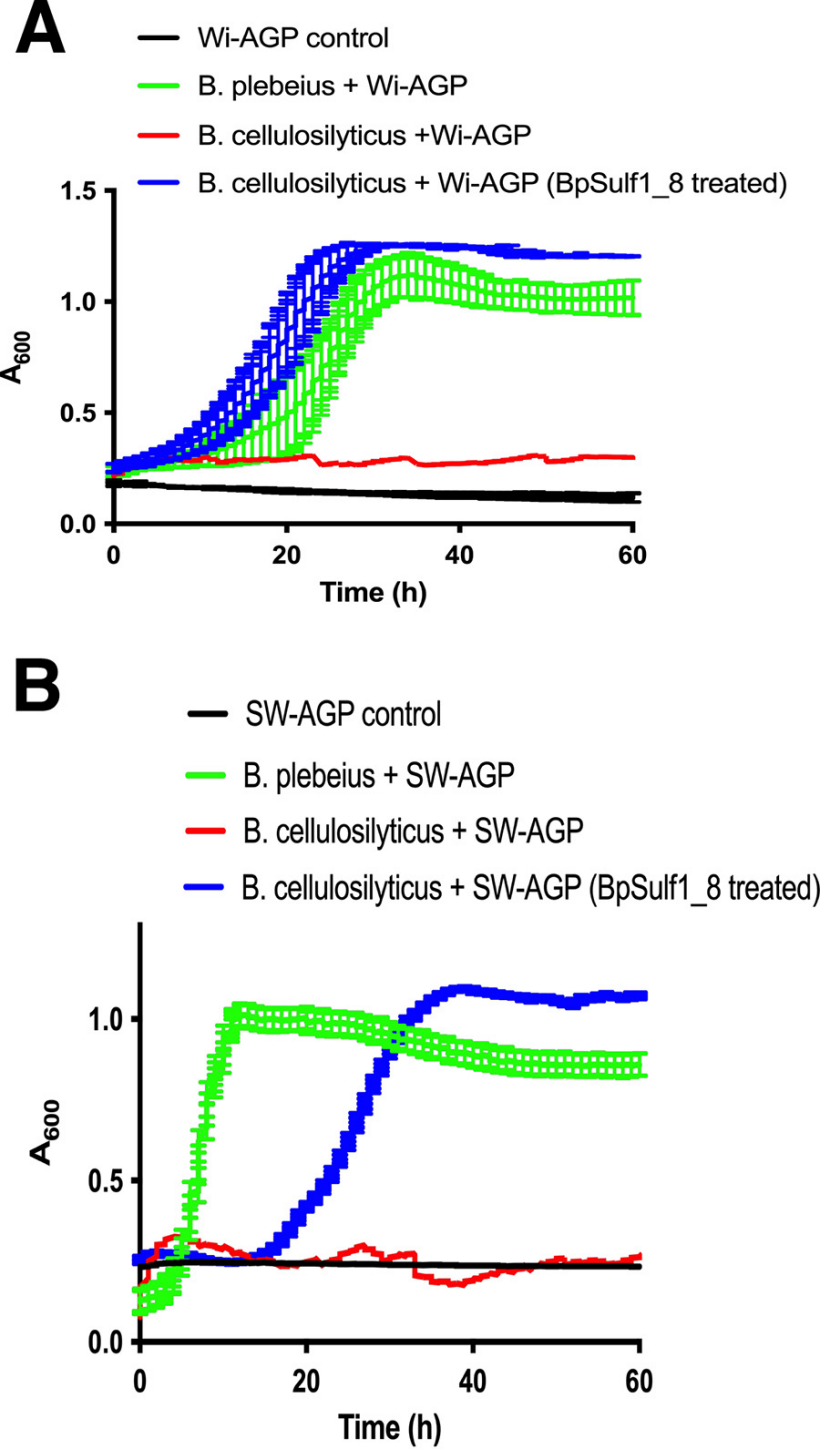
Growth of *Bacteroides* species on sulfated AGPs. Bacterial growth was assessed on SW-AGP and Wi-AGP. The *Bacteroides* species were cultured under anaerobic conditions at 37°C using an anaerobic cabinet on minimal medium containing an appropriate carbon source. The growth of the cultures was monitored by OD_600_ using a Gen5 v2.0 microplate reader. In blue, growth curves show the two AGPs had been treated with the sulfatase BpS1_8.

**FIG 6:**
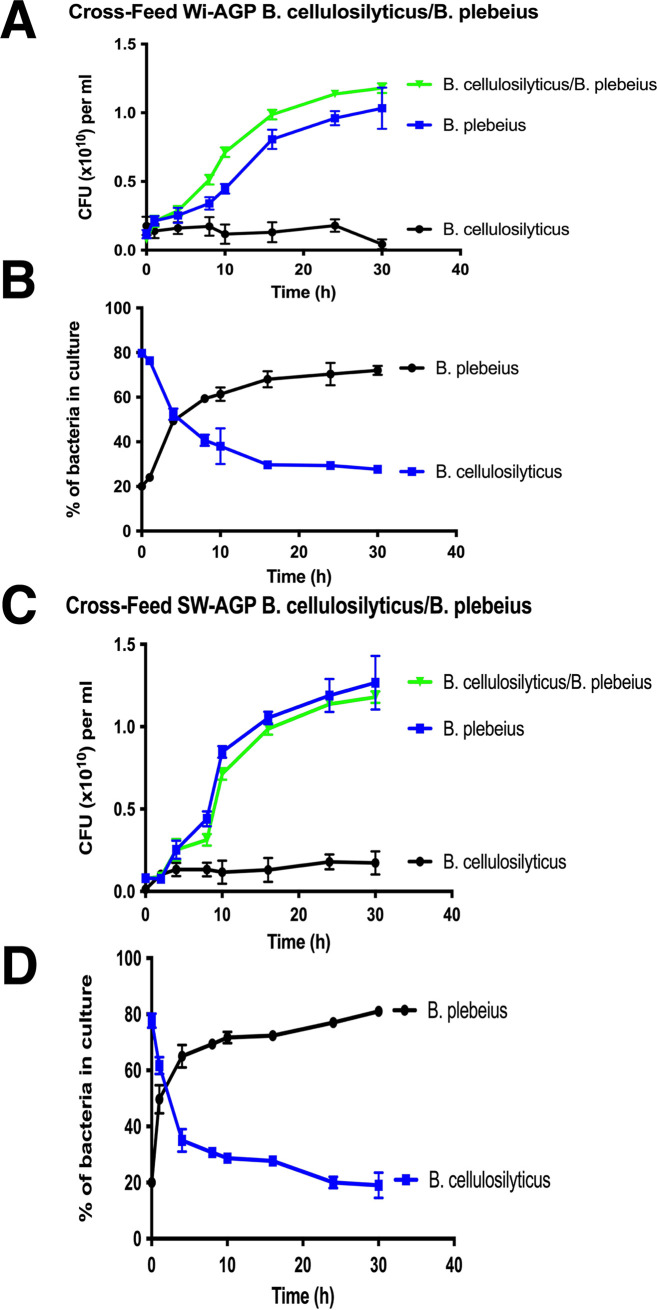
Growth profile of cocultures of *Bacteroides* species on sulfated AGPs. B. plebeius and B. cellulosilyticus strain DSM14838 were cultured on nutrient-rich (TYG) media overnight. The organisms were then inoculated at ∼10^8^ CFU per ml into minimal medium containing Wi-AGP (A and B) or SW-AGP (C and D) at 0.5% (wt/vol), either as a monoculture or in coculture. The cultures were incubated in anaerobic conditions, and at regular intervals, aliquots were removed and plated onto rich (brain heart infusion [BHI]) agar plates to determine the CFU. The ratios of the strains in the cocultures were determined by quantitative PCR with primers that amplify genomic sequences unique to each strain (see Materials and Methods for further details). (A and C) CFU for bacterial strains; (B and D) ratios of the organisms in the cocultures. Error bars represent the SEM of biological replicates (*n* = 3).

A highly unusual feature of PUL13 is the presence of two genes (Fig. 3) encoding putative sulfatases BACPEL00986 and BACPEL00990, defined as BpS1-8 and BpS1-14, respectively. This led us to hypothesize that one or more of these sulfatases contribute to the capacity of B. plebeius to deconstruct sulfated AGPs, explaining why the bacterium is the only known *Bacteroides* species within the HGM that can utilize SW-AGP and Wi-AGP as growth substrates. To evaluate this proposal, we expressed BpS1-8 in its active form and purified the N-terminal His-tagged enzyme by nickel ion affinity chromatography. The importance of sulfatase activity in the degradation of sulfated AGPs was demonstrated by exploring the activity of the GH43_24 exo-β-1,3-galactanase BpGH43_24 (BACPLE00335), which, by analogy with other enzymes in this subfamily (20), is predicted to target the backbone of AGPs, generating a series of decorated β-1,6-galactooligosaccharide side chains. The enzyme did not act on Wi-AGP; however, when the glycan was incubated with the putative exogalactanase in the presence of the predicted sulfatase BpS1_8, oligosaccharides were generated, showing that BpGH43_24 is likely active only on the desulfated polysaccharide (Fig. 4). To confirm this hypothesis, the activity of BpS1_8 was determined. The data (Table 2) showed that the enzyme released sulfate from 4-nitrophenyl-sulfate and Wi-AGP, demonstrating that BpS1_8 is a functional sulfatase, and the glycan, like SW-AGP, is sulfated. Microbial growth experiments provided further evidence for the importance of desulfation of Wi-AGP and SW-AGP when these glycans were used to culture *Bacteroides* species. The sulfatase BpS1_8 was incubated with Wi-AGP, and the treated glycan was assessed as a growth substrate for B. cellulosilyticus. The data, presented in Fig. 5, showed that the bacterium grew on the AGP only when pretreated with the sulfatase. These biochemical and microbial growth data demonstrate that desulfation of Wi-AGP is a prerequisite for its subsequent utilization by B. cellulosilyticus. Thus, the absence of sulfatase genes in the AGP PULs of *Bacteroides* species, other than B. plebeius, explains why these prokaryotes were unable to utilize the sulfated plant glycan. In a previous study, B. cellulosilyticus was shown to be a keystone organism that supports growth of other *Bacteroides* species on gum arabic AGP (GA-AGP) through the action of a surface endo-β-1,3-galactanase. The enzyme generated oligosaccharides from the AGP that could be imported and thus utilized by the recipient organisms (20). To explore whether B. plebeius could support the growth of other *Bacteroides* on sulfated AGPs, B. cellulosilyticus was cocultured with B. cellulosilyticus on Wi-AGP and SW-AGP, and the different organisms were quantified by quantitative PCR (qPCR) of genomic specific sequences of these bacteria. The data (Fig. 6) showed that the proportion of B. cellulosilyticus sharply declined over time on both AGPs, and, indeed, there was only a modest increase in the CFU of the bacterium. Thus, B. plebeius did not support the growth of B. cellulosilyticus on Wi-AGP or SW-AGP and, hence, did not fulfill a keystone role in the utilization of sulfated AGPs. This indicates that desulfation of Wi-AGP and SW-AGP is an intracellular event. Indeed, BpS1_8 contains a canonical type I signal peptide, which is entirely consistent with its predicted periplasmic location. Thus, the desulfation of Wi-AGP and SW-AGP occurred after transport into the bacterium, explaining why the desulfated glycans were not accessible to other *Bacteroides* species.

**TABLE 2:** Kinetics of GH145 enzymes and sulfatases

Enzyme or sulfatase	Substrate	*K_cat_*/*K_m_*
GH145 enzymes		
BT3686 WT^*a*^	Wi-AGP	1.80 × 10^2^ ± 5.6 × 10^1^^*b*^
BT3686 R333A	Wi-AGP	Inactive
BT3686 H90A	Wi-AGP	Inactive
BT3686 Y143F	Wi-AGP	Inactive
BACPLE00338 WT^*a*^	Wi-AGP	3.57 × 10^3^ ± 1.05 × 10^2^^*b*^
BACCELL00856 WT	Wi-AGP	1.18 × 10^3^ ± 6.90 × 10^1^^*b*^
BACCELL00856 H90Q	Wi-AGP	Inactive
Sulfatases		
BACPLE00986 S76C	Wi-AGP	0.35 ± 0.04^*b*^
BACPLE00986 S76C	SW-AGP	1.38 ± 0.14^*b*^
BACPLE00986 S76C	4NP-sulfate^*a*^	0.1447 ± 0.004^*c*^

*^a^*WT, wild-type enzyme; 4NP-sulfate, 4-nitrophenyl sulfate.

*^b^*Units are ml/mg^−1^ min^−1^.

*^c^*Unit is mM^−1^ min^−1^.

From a broader perspective, within the *Bacteroides* of the HGM, there are no examples, other than PUL13 in B. plebeius, of sulfatase genes in PULs that encode enzyme systems that degrade terrestrial plant polysaccharides. Sulfatase genes, however, are common in loci that orchestrate the utilization of human (e.g., mucins and glycosaminoglycans) and marine (e.g., porphyran) glycans. Thus, this ecosystem is tailored to utilize terrestrial plant glycans that are often acetylated but are not known to be sulfated. This report, showing that sulfatases are required to enable growth of *Bacteroides* species on Wi-AGP, provides evidence for terrestrial plant glycan sulfation. The precise nature of the sulfation of Wi-AGP is unknown and clearly requires further analysis. In addition to PUL1 and PUL13 in B. plebeius, which encode porphyran (27), and SW-AGP/Wi-AGP utilization systems, respectively, there are three further PULs that contain sulfatase genes, hinting that additional sulfated polysaccharides may be targeted by B. plebeius. Two of these loci, PUL14 and PUL15, encode glycoside hydrolases from the fucosidase families GH29 and GH95, which may suggest that B. plebeius can utilize the marine polysaccharide fucoidan, comprising sulfated fucose residues, which is abundant in brown algae (28). It should also be emphasized that human glucans, such as 6-sulfo-sialyl Lewis X and A, also contain sulfated fucose residues, and thus, PUL14 and/or PUL15 may target these glycans rather than marine polysaccharides. Nevertheless, the sulfatase genes in the B. plebeius PULs suggest that the bacterium is adapted to utilize at least some abundant marine glycans. The marine origin of the sulfatase encoding PULs is interesting. Porphyran is a major component of red algal species used to prepare culinary nori, an important component of sushi. It is believed that porphyran utilization, orchestrated by PUL1, was the result of horizontal transfer of the requisite genes from an ancestral porphyranolytic marine bacterium, related to the extant marine *Bacteroidetes* Zobellia galactanivorans in the HGM of the Japanese population, where sushi is an integral component of the diet (29). While it is tempting to speculate that the AGP and, possibly, sulfated fucose glycan-utilizing PULs are also derived from a marine *Bacteroidetes*, no obvious orthologous loci are present in Zobellia galactanivorans or any other sequenced marine bacterium. The evolutionary mechanisms by which B. plebeius acquired the ability to degrade sulfated AGP are currently unclear.

### The catalytic domain of GH145 rhamnosidases can contain two distinct and functional active sites.

**FIG 7:**
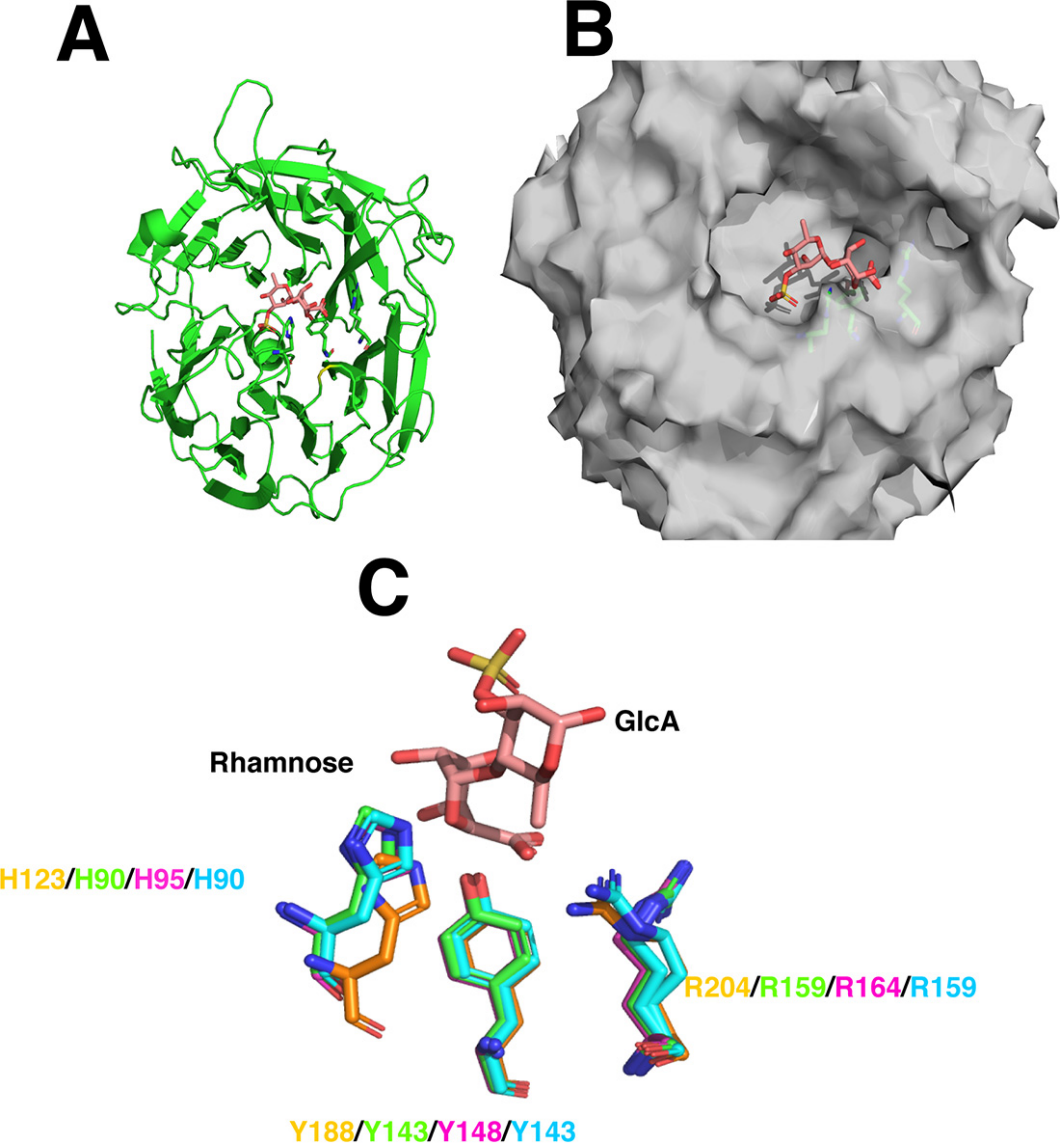
Structural conservation of the anterior active site of GH145 and PL25 polysaccharide lyases. (A) Cartoon (β-sheets depicted as broad arrows) of the β-propeller fold of the GH145 enzyme from B. cellulosilyticus (PDB ID 5MVH) viewed from the anterior surface. The ligand 3-sulfate-l-rhamnose-α-1,4-d-glucuronic acid (RhaSO_3_-GlcA) bound at the anterior catalytic center of a PL25 polysaccharide lyase (PDB ID 5UAS) is shown in stick format in salmon pink. (B) Surface representation of the broad active site pocket of the GH145 enzyme shown in panel A and the RhaSO_3_-GlcA ligand. (C) Overlay of the catalytic triad of the PL25 *Pseudoalteromonas* sp. ulvan lyase (PDB ID 5UAM; yellow) and the GH145 enzymes from B. cellulosilyticus (green), B. intestinalis (PDB ID 5MUM; magenta), and B. ovatus (PDB ID 4IRT; yellow and cyan). The ligand bound to the active site of the ulvan lyase is again shown in salmon pink.

The B. thetaiotaomicron GH145 rhamnosidase BtGH145 (BT3686), is encoded by PUL_AGPS_ (20). The crystal structure of the enzyme in complex with the reaction product, glucuronic acid, together with biochemical analysis of appropriate mutants, showed that the posterior surface houses an active site of the seven-bladed β-propeller enzyme (30). This was surprising, as the catalytic center of all other β-propeller enzymes, including several CAZymes, is located on the anterior surface (31). In addition, the central catalytic residue in the posterior active site, His48 in BtGH145, is not invariant in GH145 (Table S2 in the supplemental material), and those enzymes lacking the histidine were shown not to display rhamnosidase activity against GA-AGP. Sequence comparison of *Bacteroides* GH145 enzymes, including BpGH145 (BACPEL00338) encoded by B. plebeius PUL5, reveals that highest amino acid conservation is on the anterior surface of the enzyme (Table S2). This led to the proposal that the anterior surface also comprises a functional catalytic center at least in progenitors of GH145 and possibly in members of the current GH145 family, particularly those that do not display rhamnosidase activity against GA-AGP (20). This hypothesis is strengthened further by the high degree of structural conservation between the polysaccharide lyase belonging to PL25 and the GH145 enzymes (Fig. 7). The root mean square deviation (RMSD) between the B. thetaiotaomicron GH145 enzyme BtGH145 (PDB ID 5MUK) and the PL25 enzyme (PDB ID 5UAM) is 2.33 Å. Furthermore, the catalytic apparatus of the PL25 lyase, comprising a triad of His, Tyr, and Arg (32), is completely conserved in GH145 *Bacteroides* enzymes and is structurally equivalent in the three-dimensional structures available (Fig. 7).

10.1128/mBio.01368-21.2 TABLE S2 Alignment of GH145 *Bacteroidetes* sequences. B and D denote *Bacteroides* and *Dysgonomonas* species, respectively. The residues identified by an asterisk are conserved in all sequences. The conserved residues that are located in the anterior active site are highlighted in green; the amino acids that are conserved with the catalytic triad of the PL25 ulvan lyase are colored white in a red background. Other invariant residues are highlighted in light gray. The histidines highlighted in yellow are conserved with the catalytic residue in the posterior active site of the B. thetaiotaomicron enzyme. Download Table S2, DOCX file, 0.03 MB. Copyright © 2021 Munoz-Munoz et al. 2021 Munoz-Munoz et al. https://creativecommons.org/licenses/by/4.0/ This content is distributed under the terms of the Creative Commons Attribution 4.0 International license.

**FIG 8:**
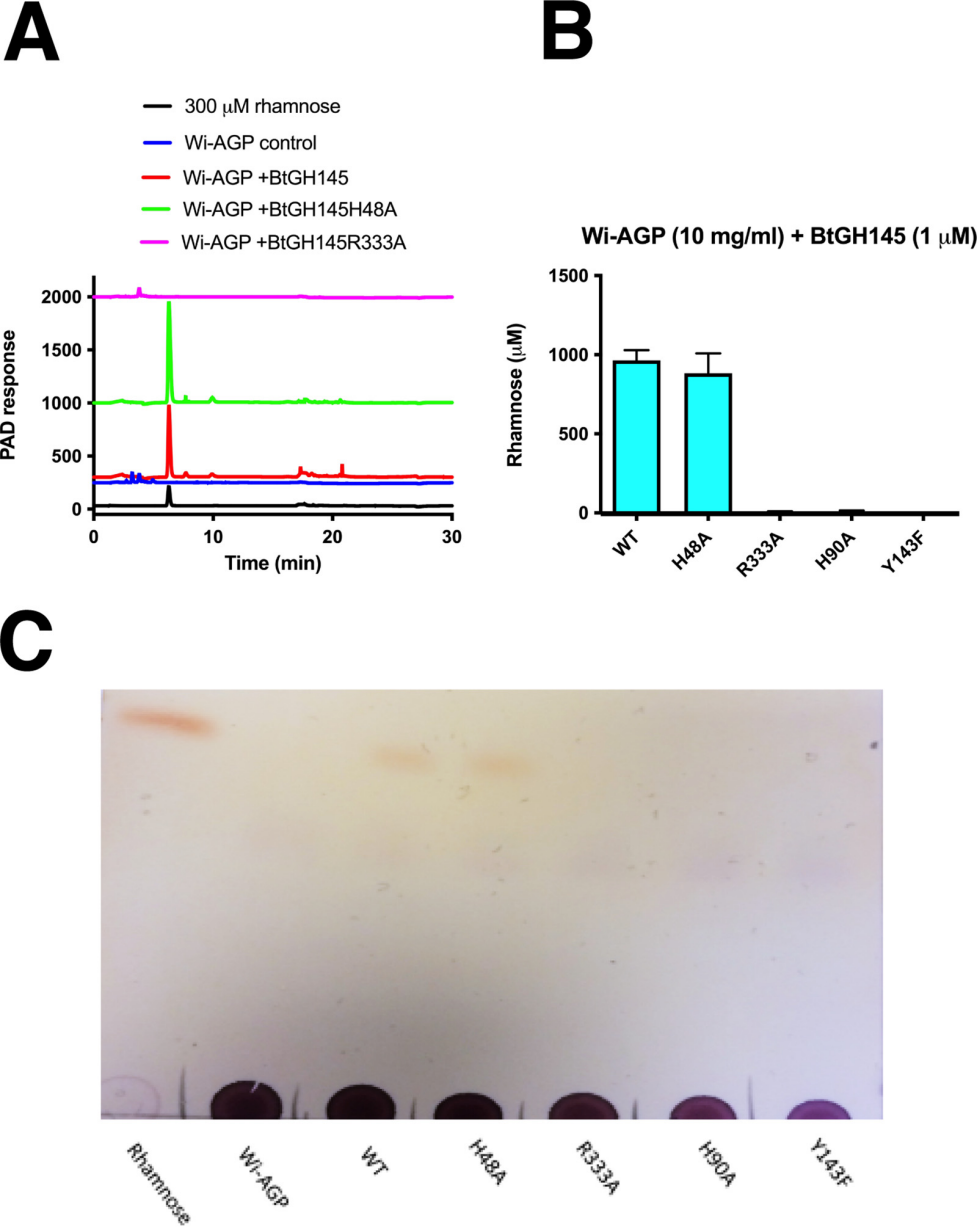
Exploring the activity of the GH145 enzyme BtGH145 against Wi-AGP. (A) Wild-type BtGH145 and mutants in which the catalytic residue in the posterior active site (H48A) and a conserved amino acid in the anterior catalytic center (R333A) were substituted with alanine and were incubated with Wi-AGP using standard conditions. After incubation for 16 h, the reaction mixtures were subjected to HPAEC using PAD to detect the reaction products. The release of rhamnose from Wi-AGP by wild type and mutants of BtGH145 were analyzed by direct biochemical quantification of the hexose sugar (B) and by TLC (C).

Based on the structural and sequence analysis of GH145, we propose that members of this family display polysaccharide lyase activity which is mediated by an active site located on the anterior surface of these enzymes. To test this hypothesis, we explored the activity of BtGH145 against Wi-AGP. The data (Fig. 8) showed that the enzyme released rhamnose, indicating it cleaves the Rha-α-1,4-GlcA linkage known to be present in this glycan (33). Significantly, rhamnose release was evident in the H48A mutant of BtGH145, which inactivates the posterior rhamnosidase (glycoside hydrolase) catalytic center that releases rhamnose from GA-AGP (30). Thus, the posterior active site that confers rhamnosidase activity does not act on Wi-AGP. This suggests that an active site in the anterior surface of the enzyme catalyzed the release of rhamnose from Wi-AGP. To evaluate this proposal, we substituted with Ala the three residues in BtGH145 (His90, Tyr143, and Arg333) located on the anterior surface that comprise the equivalent catalytic triad present in the PL25 polysaccharide lyase. The data (Table 2) showed that the three mutants were inactive against Wi-AGP, demonstrating that the release of rhamnose from this glycan was mediated by an active site on the anterior surface of the enzyme.

To evaluate further the conservation of lyase activity in GH145 enzymes, we assessed the activity of two other GH145 enzymes, BpGH145 (BACPLE00338) and BcGH145 (BACCELL00856) from B. cellulosilyticus. The two enzymes both lacked the catalytic histidine in posterior activity site (equivalent to His48 in BtGH145) and displayed no rhamnosidase activity against GA-AGP. BpGH145, and BcGH145, however, released rhamnose from Wi-AGP (Fig. 4 and Table 2). Mutation of the conserved His (His90) in the anterior surface of BcGH145 (equivalent to His90 in BtGH145) rendered the enzyme inactive against Wi-AGP (Table 2). These data, in conjunction with the conservation of the catalytic apparatus with the PL25 ulvan lyase, indicated that cleavage of rhamnose from Wi-AGP is highly likely the result of polysaccharide lyase activity. This was confirmed by further analysis of the products generated by BpGH145. The GH145 enzyme generated rhamnose and an unsaturated product, evidenced by a signal at 235 nm typical of polysaccharide lyases. The unsaturated product was sensitive to BpGH105 (BACPLE00337), a member of GH105 that contains exclusively Δ-4,5-unsaturated β-glucuronyl hydrolases and Δ-4,5-unsaturated β-galacturonyl hydrolases (34) (Fig. 4). These data show that the anterior surface of GH145 enzymes houses a catalytic center that displays polysaccharide lyase activity, irrespective of whether the posterior active site is functional.

An interesting feature of the polysaccharide lyase activity displayed by BpGH145, BcGH145, and the BtGH145 mutant H48A is that they cleave the Rha-GlcA linkage in Wi-AGP but not in GA-AGP. Although the precise nature of the Rha-GlcA targeted by the anterior lyase active site of the two GH145 enzymes is not clear, it does not comprise the simple terminal Rha-α-1,4-GlcA linkage in GA-AGP. It is probable that the anterior active site targets a sulfated substrate, likely GlcA or a sugar bound to the distal regions of the active site. It should be noted that close structural homologs of GH145 enzymes are the ulvan lyases in PL25, which catalyze Rha-GlcA linkages where the rhamnose is sulfated at O_3_ (32). As unmodified rhamnose was released from Wi-AGP by BpGH145 and BtGH145, the terminal sugar did not appear to be sulfated. The retention of the catalytic triad within the *Bacteroides* GH145 enzymes indicates that the lyase activity is a conserved feature of the family. The evolutionary rationale for retaining the lyase activity in BtGH145 is unclear, as B. thetaiotaomicron does not utilize extensively sulfated AGPs, exemplified by wine and seaweed sources of these glycans. It is formerly possible that the bacterium is able to access growth substrates that are also targeted by the lyase activity of BtGH145. Although such glycans were not evaluated here, the BtGH145 lyase activity may target AGPs where only the GlcA in the terminal Rha-GlcA capping structures is sulfated. Such glycans would be degraded by B. thetaiotaomicron and other *Bacteroides* species in the HGM. In contrast to the extensive conservation of the anterior catalytic center in GH145 enzymes, the essential catalytic histidine in the posterior active site is present in only 70% of these proteins (20) (Table S2). Indeed, in GH145 members that are not derived from the organisms in the HGM, the retention of this histidine, and thus rhamnosidase activity, is rare. This phylogenetic analysis suggests that the lyase activity is a highly conserved feature of the family and is thus a primitive feature of these enzymes, while the rhamnosidase function reflects a recent selection pressure exerted by the human diet, exemplified by GA-AGP. It would seem unlikely that the lyase activity in all the GH145 enzymes targets exclusively sulfated AGPs; however, other potential substrates for these enzymes remain to be identified.

The presence of two discrete functional active sites in a single catalytic domain is a unique feature of GH145 enzymes. Several CAZymes containing two or more catalytic modules, joined by flexible linker sequences, have been described (35). The inherent mobility between these catalytic modules allows the active sites to access different regions of a common polysaccharide substrate, enabling synergistic interactions between the different activities. This is exemplified by the degradation of acetylated xylans by enzymes displaying endo-xylanase and acetyl esterase activities (36). In GH145 enzymes, the two active sites target different AGPs, and thus, there is no obvious requirement for synergy to operate between the posterior and anterior catalytic centers.

### Conclusions.

This report shows that Wi-AGP and SW-AGP are privileged nutrients for B. plebeius within the HGM and that sulfate decorations of the glycans are responsible for their restricted use as growth substrates. The manipulation of the HGM has the potential to have significant health benefits (37, 38). The fate of exogenous commensal and probiotic strains applied to an established HGM is variable, largely unpredictable, and greatly influenced by the background microbiota (39). Studies have shown that it is possible to establish an exogenous bacterium in the HGM when porphyran, a privileged nutrient for the organism, is supplied to the ecosystem. Furthermore, transfer of the large porphyran locus (21 to 34 genes) to other organisms also conferred a substantial competitive advantage in the presence of the marine polysaccharide (40). Here, we have shown that a second sulfated polysaccharide can also act as a privileged nutrient within the HGM. We propose that the sulfation of marine polysaccharides makes these glycans excellent privileged nutrient candidates in terrestrial ecosystems, where the deployment of sulfated plant polysaccharides as growth substrates is very limited. Although engineering porphyran utilization into health-promoting organisms is an attractive strategy, transferring a 21- to 34-gene locus into these bacteria is a significant challenge. In contrast, transferring genes encoding a sulfatase, and possibly an appropriate SusC/SusD outer membrane sulfated AGP transporter, into the organism of choice may offer technical advantages that could increase the range of bacteria that can be engineered to utilize sulfated glycans such as Wi-AGP and SW-AGP.

## MATERIALS AND METHODS

### Purification of Wi-AGP.

Wi-AGP was isolated from one liter of red wine (vino d’Itialia [12% alcohol]) by gel filtration chromatography. The wine (3 liters) was concentrated by rotary evaporation and precipitated with ethanol (80% [vol/vol]). The mixture was kept at 4°C for 12 h and the resulting precipitate collected by centrifugation at 4,000 × *g* for 10 min. The precipitate was dissolved in 200 ml water, reprecipitated with 80% (vol/vol) ethanol, and the process repeated. The final mixture in water was freeze-dried, and 400 mg of the resulting powder dissolved in 50 mM sodium acetate (NaOAC), pH 5. The sample was resolved using 300 ml of Sephadex G-75 resin (Sigma; catalog no. G75120) with 50 mM NaOAC (pH 5.0) as mobile phase at a flow rate of 1 ml/min. Eluted fractions (8 ml each) were collected and analyzed by thin-layer chromatography, acid hydrolysis, and high-performance anion-exchange chromatography (HPAEC). Fractions containing Wi-AGP were pooled, dialyzed (using a VWR Visking dialysis tubing [3,500 molecular weight cutoff]), and freeze-dried.

### Cloning, expression, and purification of recombinant proteins.

DNAs encoding enzymes lacking their signal peptides were amplified by PCR using appropriate primers. The amplified DNAs were cloned into pET28a with an N-terminal His_6_ tag using NheI and XhoI restriction sites. To express an active form of the sulfatase BpS1_8, the S76C variant of the enzyme was generated. The Escherichia coli sulfatase-maturing enzyme is able to convert cysteine but not serine into the essential formylglycine residue. To express the recombinant genes encoding the AGP-degrading enzymes, Escherichia coli strains BL21(DE3) and Tuner, harboring appropriate recombinant plasmids, were cultured to mid-exponential phase in Luria-Bertani broth at 37°C. This was followed by the addition of 1 mM [strain BL21(DE3)] or 0.2 mM (Tuner) isopropyl-β-d-thiogalactopyranoside (IPTG) to induce recombinant gene expression, and the culture was incubated for a further 5 h at 37°C or 16 h at 16°C, respectively. The recombinant proteins were purified to >90% electrophoretic purity by immobilized metal ion affinity chromatography using Talon, a cobalt-based matrix, and eluted with 100 mM imidazole, as described previously (20).

### Mutagenesis.

Site-directed mutagenesis was conducted using the PCR-based QuikChange site-directed mutagenesis kit (Strategene) according to the manufacturer’s instructions, using the appropriate plasmid encoding BtGH145, BpGH145, BcGH145, or BpS1_8 as the template and appropriate primer pairs.

### CAZyme and sulfatase assays.

Spectrophotometric quantitative assays for α-l-rhamnosidase activity were monitored by the continuous formation of NADH at *A*_340_ _nm_ using an extinction coefficient of 6,230 M^−1 ^cm^−1^, with an appropriately linked enzyme assay system. The assays were adapted from the Megazyme International assay kit (product code K-rhamnose). The standard reaction conditions were supplemented with 1 mM NAD+ and an excess of l-rhamnose dehydrogenase. The dehydrogenase oxidases released rhamnose to l-rhamnose-1,4-lactone with the concomitant reduction of NAD+ to NADH. The concentrations of Wi-AGP and SW-AGP ranged from 0 to 10 mg ml^−1^. As the assays gave a linear relationship between rate and substrate concentration, only *k*_cat_/*K_m_* could be determined and not the individual kinetic parameters.

Spectrophotometric quantitative assays were deployed to measure the sulfatase activity of Bacple_00986 (BpSulf1_8) acting on SW-AGP, Wi-AGP, and 4-nitrophenyl sulfate (PNP-sulfate). For PNP-sulfate, the enzymatic assays were done in a continuous assay, monitoring the release of PNP by the enzyme and quantifying the activity at a wavelength of 400 nm. The product was quantified using a molar extinction coefficient of 10,500 M^−1 ^cm^−1^. When the polysaccharides were used as the substrates for the enzyme, we used the sulfate assay kit from Bioassay Systems (product code DSFT-200). The kit quantifies the formation of insoluble barium sulfate from barium chloride and the sulfate released by the substrate in polyethylene glycol. The turbidity measured at 600 nm is directly proportional to sulfate levels in the sample. This kit employed a stopped assay where different polysaccharide concentrations were incubated with the enzyme, aliquots were taken at different times, and the reaction was stopped by incubation at 100°C for 10 min. The aliquots were incubated with the working reagent prepared as the manufacturer indicated in the kit for 10 min, and absorbance at 600 nm was determined. To calculate the concentration of sulfate released in milligrams per milliliter, we performed a calibration curve with the same kit to ensure that the sulfate groups released fall within the curve range. We varied the polysaccharide concentrations from 0 to 10 mg/ml. As the assays gave a linear relationship between rate and substrate concentration, only *k_cat_*/*K_m_* could be determined and not the individual kinetic parameters.

The mode of action of enzymes was determined using HPAEC or thin-layer chromatography (TLC), as appropriate. In brief, aliquots of the enzyme reactions were removed at regular intervals and, after boiling for 10 min to inactivate the enzyme and centrifugation at 13,000 × *g*, the amount of substrate remaining or product produced was quantified by HPAEC using standard methodology. The reaction substrates and products were bound to a Dionex CarboPac PA100 column and glycans eluted with an initial isocratic flow of 100 mM NaOH and then a 0- to 200-mM sodium acetate gradient in 100 mM NaOH at a flow rate of 1.0 ml min^−1^, using pulsed amperometric detection or absorbance at 235 nm. In TLC assays of enzyme activity, 5 μl of each sample was spotted onto silica plates and resolved in butanol/acetic acid/water at a concentration of 2:1:1 and carbohydrate products detected by spraying with 0.5% orcinol in 10% sulfuric acid and heating to 100°C for 10 min. All reactions were carried out in 20 mM sodium phosphate buffer, pH 7.0, with 150 mM NaCl at 37°C (defined as standard conditions) and performed in at least technical triplicates.

### Growth of *Bacteroides*.

*Bacteroides* spp. were routinely cultured under anaerobic conditions at 37°C using an anaerobic cabinet (Whitley A35 workstation; Don Whitley) in culture volumes of 0.2, 2, or 5 ml of TYG (tryptone-yeast extract-glucose medium) or minimal medium (MM) (31) containing 0.5 to 1% of an appropriate carbon source and 1.2 mg ml^−1^ porcine hematin (Sigma-Aldrich) as previously described (10). The growth of the cultures was monitored by optical density at 600 nm (OD_600_) using a Gen5 v2.0 microplate reader (Biotek).

For batch fermentation of the HGM, sterile stirred batch culture fermentation vessels (300 ml working volume) were aseptically filled with 135 ml of sterile basal nutrient medium (2 g/liter peptone water, 2 g/liter yeast extract, 0.1 g/liter NaCl, 0.04 g/liter K_2_HPO_4_, 0.04 g/liter KH_2_PO_4_, 0.01 g/liter MgSO_4_·7H_2_O, 0.01 g/liter CaCl_2_·6H_2_O, 2 g/liter NaHCO_3_, 0.5 g/liter l-cysteine HCl, 0.5 g/liter bile salts, 0.05 g/liter hemin [dissolved in a few drops of 1 M NaOH], 2 ml/liter Tween 80, 0.01 ml/liter vitamin K, and 4 ml/liter resazurin solution [0.025/100 ml]). Once in the fermentation vessels, sterile medium was maintained under anaerobic conditions by sparging the vessels with O_2_-free N_2_ overnight (15 ml/min). The temperature was held at 37°C using a circulating water bath and pH values controlled between 6.7 and 6.9 using an automated pH controller (Fermac 260; Electrolab, Tewkesbury, UK), which added acid or alkali as required (0.5 M HCl and 0.5 M NaOH). After the equilibration, all vessels were inoculated with 15 ml fecal slurry. Fecal slurry was prepared using stool from three anonymous healthy donors, which was then diluted in 1× phosphate-buffered saline (PBS) (pH 7.4) with 10% (wt/vol) dilution and mixed in a stomacher for 2 min. Batch culture vessels were set up in triplicate containing no carbon source, 5 g inulin, and 5 g Wi-AGP, respectively. Fecal slurries from stool samples were then added to the vessels. The fermentations occurred for 24 h, after which bacteria were recovered and subjected to rRNA profiling as described below.

### 16S rRNA profiling of the HGM cultured *in vitro*.

Bacterial profiling of the variable region 4 (V4) of the 16S rRNA gene was carried out by NU-OMICS (Northumbria University). Briefly, PCR was performed carried out using 1× AccuPrime Pfx SuperMix, 0.5 μM each primer (515F, GTGCCAGCMGCCGCGGTAA, and 806R, GGACTACHVGGGTWTCTAAT) and 1 μl of template DNA under the following conditions: 95°C for 2 min; 30 cycles 95°C for 20 s, 55°C for 15 s, and 72°C for 5 min with a final extension at 72°C for 10 min. One positive- and one negative-control sample were included in each 96-well plate and carried through to sequencing. PCR products were normalized using SequalPrep normalization kit (Invitrogen). The DNA was then denatured using 0.2 M NaOH for 5 min and diluted to a final concentration of 5 pM, supplemented with 15% PhiX, and loaded onto a MiSeq V2 500-cycle cartridge (Illumina). The sequences obtained were run through the GALAXY server (Mothur program; https://training.galaxyproject.org/training-material/topics/metagenomics/tutorials/mothur-miseq-sop/tutorial.html) to compare their similarity to the V4 region of rRNA of 10,000 curated sequenced bacteria, derived from the human Gut Microbiome and Earth Microbiome projects.
