# Supplementary material for: Sulfation of Arabinogalactan Proteins Confers Privileged Nutrient Status to Bacteroides plebeius
Source: mBio. 2021 Aug 3;12(4):e01368-21. doi: 10.1128/mBio.01368-21 (PMC8406133; doi:10.1128/mBio.01368-21)
Supplement: TABLE S1 [file mbio.01368-21-st001.docx]

**Supplementary Table S1: % of bacterial genera or species in cultured HGM^a^**

| Genera | Negative control | Inulin | Wi-AGP |
| --- | --- | --- | --- |
| Bacteroides | 40 | 58 | 64 |
| Bifidobacterium | 5 | 6 | 8 |
| Lachnospiraceae | 15 | 15 | 12 |
| Lactobacillus | 1 | 1 | 1 |
| Ruminococcus | 8 | 6 | 3 |
| Verrumicrobia | 5 | 8 | 4 |
| Prevotella | 8 | 2 | 6 |
| Others | 18 | 4 | 2 |

| Bacteroides sp. | Negative control | Inulin | Wi-AGP |
| --- | --- | --- | --- |
| B. thetaiotamicron | 20 | 27 | 5 |
| B. ovatus | 10 | 35 | 4 |
| B. vulgatus | 9 | 4 | 2 |
| B. fragilis | 40 | 10 | 2 |
| B. uniformis | 7 | 7 | 5 |
| B. distasonis | 5 | 4 | 0.5 |
| B. plebeius | ND | ND | 78 |
| B. cellulosilyticus | 4 | 6 | 3 |
| Others | 5 | 7 | 0.5 |

^a^The data are displayed as a bar chart in Fig. 1

^b^ND; no sequence detected
