# Supplementary material for: Sulfation of Arabinogalactan Proteins Confers Privileged Nutrient Status to Bacteroides plebeius
Source: mBio. 2021 Aug 3;12(4):e01368-21. doi: 10.1128/mBio.01368-21 (PMC8406133; doi:10.1128/mBio.01368-21)
Supplement: TABLE S2 [file mbio.01368-21-st002.docx]

**Supplementary Table S2 Alignment of GH145 Bacteroidetes sequences**

B.sp.CACC737 -----MKILYSILFILCLVSCQNLSRI-----------KE—RKIDVAKVVSDFPVD----

B.plebeius_DSM17135 -----MKRIYLFLIGLLVWSEIS---------------AQ--QLVKVGDGYSSTSVNTTV

B.salanitronis_DSM18170 -----MMKHKLLLLLLCLCGYGMA--------------YP--QLVRVGEGYSNTSVNTTV

B.graminisolvens_DSM19988 MGTFLSKRILSGTFLLCVLTSSLF--------------AQ--RLVDAGKGYSRTSVNTTV

B.dorei_DSM17855 ------------------MAFSVR--------------AQ--RLVEVGKGFSSTSVNTTV

B.vulgatus_ATCC8482 ------MRNLFLLIALCCVAFSVR--------------AQ--RLVEVGKGFSSTSVNTTV

B.zoogleoformans_ATCC33285 -MRNKAKRWTRLFVPVALGLGGCLGTLSAQSVATGFQPEV--RLVEVGKGYSRTSVNTTV

B.heparinolyticus_F0111 -MRNKAKRWLRLFVPVVLGIGSSWGTLSAQSVGIGFQPEV--RLVEVGKGYSRTSVNTTI

B.thetaiotaomicron_VPI5482 -----MKRTLLLTFLLLSVSALCF--------------SQ--KLVEVAKGYSCTSVNTTI

B.caccae_ATCC43185 -----MRRILLLLCGIMFIPVLCY--------------PQ--HLVEVGKGYSCTSVNTTV

B.xylanisolvens_APCS1XY -----MKRILLLLCGIILVPTLAC--------------SQ--RLVEVGKGYSCTSVNTTV

B.ovatus_ATCC8483 -----MKRILLLLCGIMLVPVIAC--------------SQ--HLVEVGKGYSCTSVNTTV

B.sp.M10 -----MKRILLLLCGIMLVPVIAC--------------SQ--HLVEVGKGYSCTSVNTTV

B.uniformis_DSM6597 ---MSKTDSFFIVFILIVWGIGGFPVALCAQGAAGVLPET--HLVEVGKGYSQTSVNTAV

B.oleiciplenus_YIT12058 -----MNRYIFFFFLILLTAFTLR--------------AQQARLVEVGKGYSQTSVNTTV

B.cellulosilyticus_DSM14838 -----MNRYILFFFLILSTLTTLR--------------AQQSRLVEVGNGYSQTSVNTTV

B.intestinalis_DSM17393 -----MNKRILFFFLILLVFSTLR--------------AQQSRLVEVGNGYSQTSVNTTV

B.propionicifaciens_DSM19291 MTNRDNMRNRIKLFIVLSSVFVSLSTY-----------SQ--RMVPLGLGWANNSVNTTI

D.gadei_ATCCBAA-286 -----MKIKKYLLISIILFSLSAH--------------AQ--KLIPVAPGWSNNSVNVTV

D.macrotermitis_DSM27370 ---MTKAKAYLLFIFTLIIPSIIH--------------GQ--QLIPVGEGWANNSINTTV

. : . : .::

B. sp.CACC737 F---SLLTYNEMQFVAYYDTAHRMTVASRHLGEDKW-VYKVLDSSIGWDSHNNITMKVDK

B. plebeius_DSM17135 FRNSSLVTDGDVQYISYYDADGYLTVGKRALGSTEWTLHRSQYKGNVADAHNVISMMVDG

B. salanitronis_DSM18170 FRNSPLVTDGDTQYISYYDPEGYLVLGKRQIGSAEWTLKRSQYKGNVADAHNVISMMVDG

B. graminisolvens_DSM19988 FRNNSLVTYRNTQYISYYDGDGYLVVGKRKLNSDKWQLRRSQYKGNCADAHNVISLMVDG

B. dorei_DSM17855 FRNNSIVTHGNTQYISYYDAEGWLMLGKRRLGTGEWILHRTQYKGHVKDAHNIISMMVDG

B. vulgatus_ATCC8482 FRNNSIVTHGNTQYISYYDAEGWLMLGKRRLGTGEWILHRTQYKGHVKDAHNIISMMVDG

B. zoogleoformans_ATCC33285 FRNNSVVTHGDEQYIGYYDDEGFLTLGKRTAGSDQWTLHRTQYKGNVKDAHNVISIMSDG

B. heparinolyticus_F0111 FRNNSVVTHGDEQYIGYYDGDGFLTLGKRTAGSEQWTLHRTQYKGNVEDAHNVVSIMLDG

B. thetaiotaomicron_VPI5482 FRNNSLVTHGDEQYISYYDADGYLVLGKRKLNSKQWTLHRTQYRGNVKDAHNIISIMVDG

B. caccae_ATCC43185 FRNNSLITHGDEQYISYYDAEGYLVLGKRKLNSELWTLHRTQYRGNVKDAHNIISMIIDG

B. xylanisolvens_APCS1XY FRNNSLVTHGEEQYISYYDNDGYLILGKRKLDSKQWTLHRTQYQGNVKDAHNVISMMVDG

B. ovatus_ATCC8483 FRNNSLVTHGDEQYISYYDNDGYLVLGKRKLDSEQWTLHRTQYQGNVKDAHNIISMMIDG

B. sp.M10 FRNNSLVTHGDEQYISYYDNDGYLVLGKRKLDSEQWTLHRTQYQGNVKDAHNIISMMIDG

B. uniformis_DSM6597 FRNNSLVTQGDEQYISYYDAEGFLTLGKRNLHAGQWTLHRTQYKGNVKDAHNVISMMLDG

B. oleiciplenus_YIT12058 FRNNSLVTQGDEQYISYYDADGYLILGKRKLDSDQWTLNRTQYKGNVKDAHNIISMMLDG

B. cellulosilyticus_DSM14838 FRNNSLVTQGDEQYISYYDGDGYLVLGKRKLDSDQWTLKRTQYKGNVKDAHNIISMMLDG

B. intestinalis_DSM17393 FRNNSLVTQGDEQYISYYDGDGYLVLGKRKLNSDQWTLQRTQYKGNVKDAHNIISMMLDG

B. propionicifaciens_DSM19291 FRKNSLVSHNSIQFAAYYDTDGYVVLAKRNVKDKEWQKERTQYTGNVKDAHNSISIMLDG

D. gadei_ATCCBAA-286 FRKNSLVTHNNVQFIAYYDPDGFLTLGKRNIGEDSWTISKTQYKGNVSDAHNSISIMTDG

D. macrotermitis_DSM27370 FRKNSLVTHNNTQFIAYYDPDGYMVLGKRDITSTEWSLHKSQYKGKVTDAHNSISIMVDG

* .::: . *: .*** : :..* * . . *:** ::: *

B. sp.CACC737 EGYLHLSGNMHVSPLVYYRTESPLDITTLKRIDYMTGENERYTTYPQFMDGPEGKLIFHY

B. plebeius_DSM17135 DGYLHLSFDHHGNKLNYCKSTEPGAL-TLGEKEAMTGKDEDDVTYPEFYRMPDGDLLFVY

B. salanitronis_DSM18170 EGYIHVSFDHHGHPLNYCRSVAPGSL-ELGPKEAMTGTDEQDVTYPEFYKMPDGDLIFVY

B. graminisolvens_DSM19988 DGYLHVAFDHHGHPLNYCRSVAPGSL-QLGDKEAMTGADEQNVTYPEFYKLAGGDLIFVY

B. dorei_DSM17855 DGYLHLSFDHHGHKLNYCRSIAPDTL-VLGDKEPMIGNEEEDVTYPEFHLLADGGLLFVY

B. vulgatus_ATCC8482 DGYLHLSFDHHGHKLNYCRSIAPDTL-VLGDKEPMIGNEEEDVTYPEFHLLADGGLLFVY

B. zoogleoformans_ATCC33285 DGYLHVAFDHHGHPLNYCRSLAPYSL-KLGEKEPMTGIDEHNVTYPEFYSLPGGNLLFAY

B. heparinolyticus_F0111 DGYLHVAFDHHGHPLNYCRSLAPYSL-ELGEKKPMTGIDEHNVTYPEFYLLPGGNLLFAY

B. thetaiotaomicron_VPI5482 EGYLHVSFDHHGHKLNYCRSIAPGSL-ELGDKMPMTGVDEGNVTYPEFYPLTDGDLLFVY

B. caccae_ATCC43185 EGYLHISFDHHGHKLNYCRSIAPTSL-ELGEKMPMTGVDEGNVTYPEFYSLSDGDLLFVY

B. xylanisolvens_APCS1XY EGYIHVSFDHHGHKLNYCRSIAPGSL-KLGDKIPMTGVDEGNVTYPEFYSLSGGDLLFVY

B. ovatus_ATCC8483 EGYIHVSFDHHGHPLNYCRSIAPGSL-ELGDKMPMTGVDEGNVTYPEFYPLSGGDLLFVY

B. sp.M10 EGYIHVSFDHHGHPLNYCRSIAPGSL-ELGDKMPMTGVDEGNVTYPEFYPLSGGDLLFVY

B. uniformis_DSM6597 DGYIHVAFDHHGQPLNYCRSIAPHSL-ELGEKEPMTGVDEGNVTYPEFYLLSGGDLLFAY

B. oleiciplenus_YIT12058 DGYLHVSFDHHGHKLNYCRSIAPHAL-ELGDKEQMTGVDEGNVTYPEFYPLNGGDLLFVY

B. cellulosilyticus_DSM14838 EGYLHLSFDHHGHKLNYCRSTAPYTL-ELGDKEPMTGVDEGNVTYPEFYPLNGGDLLFVY

B. intestinalis_DSM17393 DGYLHLSFDHHGHKLNYCRSTAPYTL-ELGDKEPMTGVDEGNVTYPEFYPLNGGDLLFVY

B. propionicifaciens_DSM19291 DGYLHLSWDHHGQALNYAKSIEPYSL-TMGDKEMMTTISEGNVTYPEFYKMPNGNLIFIY

D. gadei_ATCCBAA-286 DGYLHVSWDHHGHPLRYAKGIAPYSL-ELGDKQPMTGSLETNVTYPEFLKMPDGDLIFMY

D. macrotermitis_DSM27370 DGYLHVSWNHHGDPLSYAKGTMPYGL-ELTKKLPMTGISENNVTYPEFFKMGDGSLIFMY

:**:*:: : * * * . * : : * * .***:* * *:* *

B. sp.CACC737 RDGGSGNGNEIYNVYDYNTQTWSKLLDCPLTDGEGLMNAYMDGPTLGPDEYYHLLWVWRD

B. plebeius_DSM17135 RSGASGRGNLVLNRYDLKSRKWTRVQDV-LIDGENQRNAYWQ-LYMDKAGVIHLSWVWRE

B. salanitronis_DSM18170 RSGASGRGNLIMNRYDLDTHRWERVQDI-LIDGEGQRNAYWQ-LYVDEKGTIHLSWVWRE

B. graminisolvens_DSM19988 RSGSSGRGNLVMNRYSLVSRKWERVQDV-LIDGENQRNAYWQ-LYVDEAGTIHLSWVWRE

B. dorei_DSM17855 RSGASGRGNMVMNRYDVKSRKWERVQDV-LVDGENERNAYWQ-LYVDQSGTIHLSWVWRE

B. vulgatus_ATCC8482 RSGASGRGNMVMNRYDVKSRKWERVQDV-LVDGENERNAYWQ-LYVDQSGTIHLSWVWRE

B. zoogleoformans_ATCC33285 RSGSSGRGNLVLNGYDVKSRKWHRIQNV-LIDGEEQRSAYWQ-LYVDEQGTIHLSWVWRE

B. heparinolyticus_F0111 RSGSSGRGNLVLNGYDVKNRKWHRIQDV-LIDGEEQRSAYWQ-MYVDERGTIHLSWVWRE

B. thetaiotaomicron_VPI5482 RSGSSGRGNLVMNRYSLKDHKWARVQDV-LIDGEDKRNAYWQ-LYVDEKGTIHLSWVWRE

B. caccae_ATCC43185 RSGSSGRGNLVMNHYSLKEHKWSRVQDV-LIDGENKRNAYWQ-LYVDEQGTIHLSWVWRE

B. xylanisolvens_APCS1XY RSGFSGRGNLVMNRYSLKEHKWTRVQDI-LIDGENKRNAYWQ-MYVDEKGTIHLSWVWRE

B. ovatus_ATCC8483 RSGSSGRGNLVMNRYSLKEHKWTRVQDI-LIDGENKRNAYWQ-LYVDEKGTIHLSWVWRE

B. sp.M10 RSGSSGRGNLVMNRYSLKEHKWTRVQDI-LIDGENKRNAYWQ-LYVDEKGTIHLSWVWRE

B. uniformis_DSM6597 RSGSSGRGNLVMNRYSLKEKKWSRVQDV-LIDGENKRNAYWQ-LYVDELGTIHLSWVWRE

B. oleiciplenus_YIT12058 RSGSSGRGNLVMNRYSVKEKKWSRVQDI-LIDGEDKRNAYWQ-LYVDEQGTIHLSWVWRE

B. cellulosilyticus_DSM14838 RSGSSGRGNLVMNHYSVKEKKWNRVQDV-LIDGEDQRNAYWQ-LYVDEQGTIHLSWVWRE

B. intestinalis_DSM17393 RSGSSGRGNLVMNRYSVKEKKWYRVQDV-LIDGEDQRNAYWQ-LYVDEQGTIHLSWVWRE

B. propionicifaciens_DSM19291 RDGSSGRGNLVLNAYDLQTREWTQVQNN-LIDGENARNAYWQ-CYVDKQGTIHLSWVWRE

D. gadei_ATCCBAA-286 RDGQSGRGNLVMNRYNQKSKTWTQIQKN-FIDGENQRNAYWQ-SCIDDKGIIHLSWVWRE

D. macrotermitis_DSM27370 RDGQSGQGNLVINKYDCKSKIWSQIQNN-LIDGENKRNAYWQ-ACVDNKGAIHISWVWRE

*.* **.** : * *. . * .: . : *** .** : :. *: ****:

B. sp.CACC737 TYDCSTNHHLSYAKSKDL-VHWVNIADKSVELPITISDTCTWVDPIPVKGGIINGAAKMG

B. plebeius_DSM17135 TWMVETNHDLCYAYSPDEGKTWYKSTGEKYTLPICKDN-AEYACRIPQNSELINQTSMST

B. salanitronis_DSM18170 TWMVETNHDLCYASSPDGGKTWYTSAGEPYTLPIRKGN-AEYAWHIPQNSELINQTSMCT

B. graminisolvens_DSM19988 NWLVETNHDLCYARSRDGGKTWEKSNGETYALPINATN-AEYACRIPQNSELINQTSMSA

B. dorei_DSM17855 TWHVETNHDLCYARSFDGGKTWYKTNGQKYELPIRLGN-AEYACRIPQNAELINQTSMST

B. vulgatus_ATCC8482 TWHVETNHDLCYARSFDGGRTWYKTNGQKYELPIRLGN-AEYACRIPQNAELINQTSMST

B. zoogleoformans_ATCC33285 TWHVETNHDLCYARSFDNGVTWYKSDGEKYELPIRAAN-AEYACRIPQESELINQTGMSA

B. heparinolyticus_F0111 TWHVETNHDLCYARSFDNGVTWYKSDGEKYELPIRAAN-AEYVCRIPQESELINQTGMSA

B. thetaiotaomicron_VPI5482 TWQVETNHDLCYARSFDNGVTWYKSDGEQYKLPITASN-AEYACRIPQNSELINQTSMSA

B. caccae_ATCC43185 TWQVETNHDICYARSFDNGVTWYKSSGEQYELPIKLSN-AEYACRLPQNSELINQTSMSA

B. xylanisolvens_APCS1XY SWHVETNHDICYARSFDNGVTWYKSSGEQYELPIKLSN-AEYACRLPQNCELINQTSMSA

B. ovatus_ATCC8483 TWHVETNHDICYARSFDNGVTWYKTSGERYELPIKLSN-AEYACRLPQNCELINQTSMSA

B. sp.M10 TWHVETNHDICYARSFDNGVTWYKTSGERYELPIKLSN-AEYACRLPQNCELINQTSMSA

B. uniformis_DSM6597 TWHVETNHDLCYARSFDNGVTWYKANGKKYDLPIRLGN-AEYACRIPQNSELINQTSMSA

B. oleiciplenus_YIT12058 TWHVETNHDLCYARSFDNGVTWYKANGKQYDLPIRYNN-AEYACRIPQNSELINQTSMSA

B. cellulosilyticus_DSM14838 TWHVETNHDLCYARSYDNGVTWYKANGKKYDLPIRYNN-AEYACRIPQNSELINQTSMSA

B. intestinalis_DSM17393 TWHVETNHDLCYARSYDNGVTWYKANGKKYDLPIRYNN-AEYACRIPQNSELINQTSMSA

B. propionicifaciens_DSM19291 SWLVETNHDMCYARSRDGGATWTNSKDELYETPITLVS-AEYAARIEQDSELINQTSMTT

D. gadei_ATCCBAA-286 TADVATNHDLCYARSSDGGVSWENSKGEKYSLPITVAK-SEIACPIPQNSELINQTSMVT

D. macrotermitis_DSM27370 TPDVATNHDLCYARSSDGGITWENSKSEKYSLPITATT-AEIVCHIPQNSELINQTSMST

. *** :.** * * * . .: ** . . : . :** :.

B. sp.CACC737 FDSNNNLLITYHKYDEAGNTQGYITRYENNGWKIVPLSKWNYRWNFQGGGS----IGKGY

B. plebeius_DSM17135 DAEGHPYIVTYWRDADSEVPQYRLVWNDGKGWQNRQIMNRSQGFSLKGGGTKMIPISRPR

B. salanitronis_DSM18170 DAEGHPYIVTYWREPDSDVPQYRVVWNDGTRWNMREVMKRTLAFSLKGGGTKMIPIARPR

B. graminisolvens_DSM19988 NKEGHPFIATYWRSANSNVPQYRLVWFDGKQWRQQQVTQRVTPFSLSGGGTKMIPIARPR

B. dorei_DSM17855 DAGGHPYIATYWRNPDSDVPQYRIVWHDGVNWHNRQVSERKTPFSLKGGGTKMIPMSRPR

B. vulgatus_ATCC8482 DAGGHPYIATYWRNPDSDVPQYRIVWHDGVNWHNRQVSERKAPFSLKGGGTKMIPMSRPR

B. zoogleoformans_ATCC33285 DAEGHPYIASYWRDADSDIPQYRVVWHDGQKWNSRQVSSRHTPFSLKGGGTKMIPISRPR

B. heparinolyticus_F0111 DAGGNPYIASYWRDADSDVPQYRVMWHDGQKWNIRQVSSRRTPFSLKGGGTKMIPVSRPR

B. thetaiotaomicron_VPI5482 DAGGNPYIATYWRSSDSEVPQYRIVWNDGKTWHNRQVTDRKTPFTLKGGGTKMIPVARPR

B. caccae_ATCC43185 DAGGNPYIATYWRDPDSNIPQYRIVWNDGKVWHHRQVTDRKTPFTLKGGGTKMIPIARPR

B. xylanisolvens_APCS1XY DAEGNPYIATYWRDSDSDVPQYRIVWNDGKVWHQRQITDRKTPFTLKGGGTKMIPIARPR

B. ovatus_ATCC8483 DAGGNPYIATYWREPNSDVPQYRIVWNDGKMWHQRQITDRQTPFTLKGGGTKMIPIARPR

B. sp.M10 DAGGNPYIATYWREPDSDVPQYRIVWNDGKMWHQRQITDRRTPFTLKGGGTKMIPIARPR

B. uniformis_DSM6597 DAGGNPYIASYWRDPDSDVPQYRIVWHDGQMWHSRQVSGRTTPFSLKGGGTKMIPMARPR

B. oleiciplenus_YIT12058 DASGNPYIATYWRNPDSEVPQYRIVWHDGEMWHNRQVSDRHTSFSLKGGGTKMIPIARPR

B. cellulosilyticus_DSM14838 DASGNPYIATYWRDPDSEVPQYRIVWHDGQMWHNRQVSNRHTPFSLKGGGTKMIPIARPR

B. intestinalis_DSM17393 DASGNPYIATYWRDPDSDVPQYRIVWHDGQMWQNRQVSDRHTPFSLKGGGTKMIPIARPR

B. propionicifaciens_DSM19291 DAKGNPYIATYWSNKETGIPQYHIVYNDGKEWNTLALNFRQTSFSLKGGGTKKIPISRPQ

D. gadei_ATCCBAA-286 DAKGNPYIATYWREQDSDIPQYHIVYYTGKKWNDLNLGFRKTPFSLKGMGTKRIPISRPQ

D. macrotermitis_DSM27370 DNEGKPYIASYWRTENSDVPQYHVVYHDGKEWHDLNLGFRNTPFSLKGHGTKRIPISRPQ

.: : :*. :: .* : . *. : . :.:.* *: :..

B. sp.CACC737 VVIG---------APFCQDLGVLKINFEHIKE-GKGYWLVDEKTLSPMQEYVTGKSEGYG

B. plebeius_DSM17135 IAVD--KG--KAYFVFRDAERGSKVSMAYTDDVKSGKWQVKDLTDFSV--------DAWE

B. salanitronis_DSM18170 IVVD--GN--KACFVFRDAERGSKVSMAYTDDLRKGEWKVKDLTGFSV--------EAWE

B. graminisolvens_DSM19988 LVINEKGGKCKVAYIYRDEERGSKVSMAVTDNVESGKWMFSDLTDFSV--------EAWE

B. dorei_DSM17855 MVVD--NG--EVYYLFRDQERGSKVSIYYTKDVQFGEWRVKDLTDFAV--------NAWE

B. vulgatus_ATCC8482 MVVD--NG--EVYYLFRDQERGSKVSMYYTKDIQFGEWHVKDLTDFAV--------NAWE

B. zoogleoformans_ATCC33285 IVVD--KG--EVFYLFRDEERGSRVSVAHTKDVTTGTWSICDLTDFSV--------EAWE

B. heparinolyticus_F0111 IVVD--KG--EVFYLFRDVERGSRVSVAHTEDVVTGRWNICDLTGFSV--------DAWE

B. thetaiotaomicron_VPI5482 IVVE--DG--EIFYIFRDEERGSRVSMAHTADVANGKWIVTDLTDFSV--------DAWE

B. caccae_ATCC43185 IVVG--GG--EVFYIFRDEERGSCVSIAHATDLAISQWTITDLTDFSV--------DAWE

B. xylanisolvens_APCS1XY IVVE--GG--EVFYIFRDEERGSRVSMAHASDVGISKWTITDLTDFSV--------DAWE

B. ovatus_ATCC8483 IVVE--GG--EVFYIFRDEERGSRVSMAHATDVGTSKWTITDLTDFSV--------DAWE

B. sp.M10 IVVE--GG--EVFYIFRDEERGSRVSMAHTTDVGTSKWTITDLTDFSV--------DAWE

B. uniformis_DSM6597 IVVD--GG--EIFYVFRDEERGSKVSLAHATDVANSKWSISDLTDFTV--------GAWE

B. oleiciplenus_YIT12058 IVVE--GG--EIFYIFRDEERGSRVSMAHAPAVGTGDWTFTDLTDFPV--------DAWE

B. cellulosilyticus_DSM14838 IVVD--GG--EIFYIFRDEERGSRVSLAHAAAVGVGKWTFTDLTNFPV--------DAWE

B. intestinalis_DSM17393 IVVD--GG--EIFYIFRDEERGSRVSMAHAQAVGTGKWTITDLTDFAV--------DAWE

B. propionicifaciens_DSM19291 LVSANNGK--SLYLLFRDEERNTKASMAICKNIKKPRWEIKDLTDFEL--------GSWE

D. gadei_ATCCBAA-286 VVAKVKGKSTELYLLFRDEERGEKASVAICKDIKKNKWEISDLTDVSL--------GSWE

D. macrotermitis_DSM27370 IIAKQEREKTSLYLLFRDEERGEKASVAICKDLNNKKWEIKDLTNFPV--------NAWE

: : : .. * . : * : .:

B. sp.CACC737 PTTETEVPLVMNKCIVPDAGNRKTADCQYFLEWYSFPANRDTIRTTVKAVPSMLQVVERK

B. plebeius_DSM17135 PSYDTELWKTHQK-----------------LHVFVQETHQGDGEKVKASEATPVYVLEVN

B. salanitronis_DSM18170 PSLDTELWKQQKR-----------------LHLFVQTAYQGDGEKTVEKEPTPVYVLEVD

B. graminisolvens_DSM19988 PSHDTELWKSRRQ-----------------LHLFVQKTAQGDGEKTVEQEPTSVYVLEKI

B. dorei_DSM17855 PSHDTELWKMKKQ-----------------LHIYVQDTRQGDGEKQVETEPQMVYVLEL-

B. vulgatus_ATCC8482 PSHDTELWKMKKQ-----------------LHIYVQDTRQGDGEKQVETEPQMVYVLEL-

B. zoogleoformans_ATCC33285 PSHDTELWKRKRL-----------------LHLFVQKTMQGDGERTVEIAAQPVYVLEVL

B. heparinolyticus_F0111 PSHDTELWKQKRL-----------------LHLFVQKTMQGDGEQAVEIAAQPIYVLEVL

B. thetaiotaomicron_VPI5482 PSHDTELWKKQRK-----------------LNLFVQHTCQGDGERTAEIEPQMIYVLEAN

B. caccae_ATCC43185 PSHDTELWKKQRK-----------------LHLFVQHTRQGDGERMAEIEPQMVYVLEMD

B. xylanisolvens_APCS1XY PSHDTELWKKQRK-----------------LHLFVQHTRQGDGERTAEIDPQMVYVLETD

B. ovatus_ATCC8483 PSHDTELWKKQRK-----------------LHLFVQHTRQGDGERTAEIEPQMIYVLETN

B. sp.M10 PSHDTELWKKQRK-----------------LHLFVQHSRQGDGERTAEIEPQMIYVLETN

B. uniformis_DSM6597 PSHDTELWKSRKR-----------------LHLFVQHAKQGDGERVVEFAPQSVYVLEVI

B. oleiciplenus_YIT12058 PSHDTELWKQKRR-----------------LHLFVQHTKQGDGERMVEFAPQPVYVLEVN

B. cellulosilyticus_DSM14838 PSHDTELWKQKRR-----------------LHLFVQHTKQGDGERRVEFAPQPVYVLEVV

B. intestinalis_DSM17393 PSHDTELWKQKRR-----------------LHLFVQHTKQGDGERMVEFAPQPVYVLEVN

B. propionicifaciens_DSM19291 PSFDTELWKEKRL-----------------LHLFIQSTTQVDGEGQADVEPSMVYVLEVD

D. gadei_ATCCBAA-286 PSFDTELWREKGL-----------------LHVFIQKVEQVDGEGKADVEPQMVNVLEVE

D. macrotermitis_DSM27370 PSYDTELWRTQQK-----------------LHVFVQKVEQIDGEKTADVTSQPVQVLEVE

*: :**: * : . . : *:*

B. sp.CACC737 LKW--

B. plebeius_DSM17135 -----

P. salanitronis_DSM18170 MGKK-

B. graminisolvens_DSM19988 KM---

B. dorei_DSM17855 -----

B. vulgatus_ATCC8482 -----

B. zoogleoformans_ATCC33285 E----

B. heparinolyticus_F0111 K----

B. thetaiotaomicron_VPI5482 TNTKK

B. caccae_ATCC43185 MNTKK

B. xylanisolvens_APCS1XY MNINK

B. ovatus_ATCC8483 MDTNK

B. sp.M10 MDTNK

B. uniformis_DSM6597 R----

B. oleiciplenus_YIT12058 NND--

B. cellulosilyticus_DSM14838 KPKGD

B. intestinalis_DSM17393 N----

B. propionicifaciens_DSM19291 KF---

D. gadei_ATCCBAA-286 -----

D. macrotermitis_DSM27370 INIK-

B. and D. denote *Bacteroides* and *Dysgonomonas* species, respectively. The residues identified by an asterisk are conserved in all sequences. The conserved residues that are located in the anterior active site are highlighted in green, the amino acids that are conserved with the catalytic triad of the PL25 ulvan lyase are coloured white in a red background. Other invariant residues are highlighted in light grey. The histidines highlighted in yellow are conserved with the catalytic residue in the posterior active site of the *B. thetaiotaomicron* enzyme.
